# Optimisation of Healthy-Lipid Content and Oxidative Stability during Oil Extraction from Squid (*Illex argentinus*) Viscera by Green Processing

**DOI:** 10.3390/md19110616

**Published:** 2021-10-30

**Authors:** Alicia Rodríguez, Marcos Trigo, Santiago P. Aubourg, Isabel Medina

**Affiliations:** 1 Department of Food Science and Chemical Technology, Faculty of Chemical and Pharmaceutical Sciences, University of Chile, C/Santos Dumont, 964, Santiago 8380000, Chile; arodrigm@uchile.cl; 2 Department of Food Technology, Marine Research Institute (CSIC), c/E. Cabello 6, 36208 Vigo, Spain; mtrigo@iim.csic.es (M.T.); medina@iim.csic.es (I.M.)

**Keywords:** *Illex argentinus*, viscera, green extraction, oil, yield, ω3 PUFA, rancidity, polyene index, RSM optimisation

## Abstract

Green extraction was applied to Argentinean shortfin squid (*Illex argentinus*) viscera, consisting of a wet pressing method including a drying step, mechanic pressing, centrifugation of the resulting slurry, and oil collection. To maximise the oil yield and ω3 fatty acid content and to minimise the oil damage degree, a response surface methodology (RSM) design was developed focused on the drying temperature (45–85 °C) and time (30–90 min). In general, an increase of the drying time and temperature provided an increase in the lipid yield recovery from the viscera. The strongest drying conditions showed a higher recovery than 50% when compared with the traditional chemical method. The docosahexaenoic and eicosapentaenoic acid contents in the extracted oil revealed scarce dependence on drying conditions, showing valuable ranges (149.2–166.5 and 88.7–102.4 g·kg^−1^ oil, respectively). Furthermore, the values of free fatty acids, peroxides, conjugated dienes, and ω3/ω6 ratio did not show extensive differences by comparing oils obtained from the different drying conditions. Contrary, a polyene index (PI) decrease was detected with increasing drying time and temperature. The RSM analysis indicated that optimised drying time (41.3 min) and temperature (85 °C) conditions would lead to 74.73 g·kg^−1^ (oil yield), 1.87 (PI), and 6.72 (peroxide value) scores, with a 0.67 desirability value.

## 1. Introduction

Marine products are reported to provide highly nutritional constituents to human diet [1]. On the basis of their chemical composition, marine lipids differ from lipids from other plant and animal sources in that they contain a wider range of fatty acids (FAs), longer-chain FAs, and a larger proportion of highly unsaturated FAs, particularly ω3 FAs, such as eicosapentaenoic (EPA) and docosahexaenoic (DHA) acids [2]. Interestingly, clinical and epidemiological studies have associated EPA consumption with low prevalence of coronary, circulatory, and inflammatory diseases [3], while DHA has been related to foetal development, prevention of neurodegenerative diseases, and correct functioning of the nervous system and visual organs in the foetus [4].

In order to produce marine fats and oils, a wide range of methods have been used, such as physical fractionation [5], chemical solvent extraction [6], supercritical fluid extraction [7], pH adjustment [8], and enzymatic hydrolysis [9]. In the last decades, there has been an increased focus on green methods in an attempt to reduce the risk of chemical exposure to humans and the environment. Among them, the wet pressing method, including cooking, pressing, decantation, and centrifugation, is the most commonly used method for oil production on an industrial scale [10,11,12]. However, the drastic temperature and pressure conditions used for final oil release may partially decrease the polyunsaturated fatty acids’ (PUFAs’) quality [13]. Consequently, alternative methods have shown the need for carrying out softer extraction conditions including partial drying, pressing, and centrifugation [14,15,16].

Marine by-products (blood, viscera, heads, bellies, bones, skin, etc.) constitute a main concern for current fishery management policies and legislations [17]. However, marine by-products represent a significant source of main constituents like proteins, vitamins, minerals, and lipids, in addition to minor components, such as amino acids, enzymes, collagen, pigments, chitin, and other bioactive compounds [18,19]. Consequently, a profitable employment of such compounds has been described for human nutrition, as well as for nutraceutical, pharmaceutical, and cosmeceutical industries [20,21]. Among such by-products, viscera represent an important part of the fish and invertebrate mass, with their valorisation allowing a value increase of this kind of by-product as an important source of ω3 PUFAs [22,23].

Cephalopod species are an important economic resource for global fisheries. Among squid species, Argentinean shortfin squid (*Illex argentinus*) catches have gradually increased in the last decade due to a growing market demand and an expansion of fisheries into new fishing grounds and deeper waters [24]. During the commercial processing of this species, high quantities of by-products are negated to improve their preservation, reduce the shipping weight, and increase the quality of the main product. The current study focused on the oil extraction from viscera components resulting from the commercialisation of this cephalopod species. As expressed in the Materials and Methods section, a green extraction methodology was applied, this consisting of a wet pressing method without previous cooking but including a preliminary partial drying step. In order to maximise the oil yield and the content on ω3 PUFAs (namely, DHA and EPA) and to minimise the oil damage degree (hydrolysis and oxidation development), a response surface methodology (RSM) design was carried out.

## 2. Results and Discussion

### 2.1. Moisture Loss during the Drying Process and Pressed Liquor Yield

Starting viscera showed a moisture content of 647.3 ± 4.4 g·kg^−1^ viscera (Table 1), which can be considered similar to studies concerning viscera by-products in other cephalopod species, such as mitre squid (*Loligo formosana*) [25], giant squid (*Dosidicus gigas*) [26], cuttle fish (*Sepia officinalis*) [27], or octopus (*Octopus vulgaris*) [28]. The drying process led to varying values of moisture loss, with the values being included in a wide range (1.7–51.1 g·kg^−1^ viscera; Table 1). Thus, the moisture content in dried viscera was included in the 613.9–642.9 g·kg^−1^ viscera range. It could be observed that an increase of the drying conditions (i.e., time and temperature) led to an increase of water loss as a result of the heating effect on viscera samples. Notably, the employment of the strongest condition tested (85 °C for 90 min) provided a moisture loss higher than 50 g·kg^−1^ viscera. In contrast, samples corresponding to the drying condition including the lowest temperature (i.e., 45 °C) or the lowest time (i.e., 30 min) did not overpass a loss level of 10 g·kg^−1^ viscera. The current results agree with previous research showing a great dependence of moisture loss on drying conditions for different kinds of marine species [29,30].

As for moisture loss, the yield of the resulting pressed liquor showed a marked effect of both preliminary drying parameters (Table 1). Values of the recovered slurry were included in the 279.4–641.4 g·kg^−1^ viscera range. An increase of the drying time and temperature conditions led to higher values of pressed liquor in most cases, so that the highest levels (*p* < 0.05) were found in samples corresponding to 65 and 85 °C when heated for 60 and 90 min.

### 2.2. Oil Yield from Squid Viscera

Starting viscera showed an oil content of 111.3 ± 6.3 g·kg^−1^ viscera (Table 2); this value can be considered as remarkably higher than values reported for edible parts of cephalopod species in general [28,31]. Compared with the current squid viscera, Toyes-Vargas et al. [26] indicated a higher level (199.8 g·kg^−1^ viscera) for giant squid (*D. gigas*) viscera, while a lower value was detected by Kacem et al. [27] in cuttlefish (*Sepia officinalis*) viscera (stomach, intestines, and pyloric caeca) during a seasonal study (5.8–40.2 g·kg^−1^ viscera). Furthermore, Saito et al. [32] obtained a lipid content (g/100 g tissue) of 15.7–17.9 for liver and 1.0–1.4 for gonad in the case of Humboldt squid (*D. gigas*), while Hayashi and Kishimura [33] indicated a great lipid level (g/100 g tissue) in liver (56.9) and a marked difference between ovary (13.9) and testis (1.5) levels in the squid *Berryteuthis magister*.

The oil yield obtained from viscera in the current study showed a marked dependence on the previous drying conditions employed (Table 2). Thus, a high variability was observed (0.5–84.1 g·kg^−1^ viscera) as in the case of the moisture loss value (Table 1). In most cases, increasing the previous drying time and temperature led to a higher recovery of lipids from viscera. Notably, three of the softest treatment conditions (i.e., 45 °C/30 min, 45 °C/60 min, and 65 °C/30 min) hardly led to oil separation after centrifugation of the pressed slurry, so that very low oil recoveries were obtained in such cases. Taking into account the lipid content of the starting viscera (111.3 ± 6.3 g·kg^−1^ viscera), a higher recovery than 50% (i.e., 53–76% range) was obtained by applying the previous drying conditions including heating at 85 °C at any of the drying times tested, as well as by applying a drying treatment corresponding to 65 °C for 60 min. Lipids not recovered at the top layer after centrifugation are likely to be retained in the solid fraction resulting from the pressing step or remain in the water emulsion resulting from the centrifugation step. According to previous research, polar lipids, i.e., phospholipids, are likely to be retained in such kinds of emulsion fractions as they are reported to have emulsifier properties [18,34].

As in the current research, Chantachum et al. [14] found a marked effect of heating temperature (75–95 °C) and time (10–30 min) during oil extraction from skipjack tuna (*Katsuwonus pelamis*) heads by a wet reduction method. In this study, optimum conditions for the separation of crude oil involved previous heating of samples at 85 °C for 30 min, followed by wet pressing followed by centrifugation. Pudtikajorn and Benjakul [11] extracted oil from skipjack tuna (*K. pelamis*) eyeballs by the wet rendering method at 121 °C for different holding times (5–60 min). As a result, oil yield increased as heating time increased up to 20 min, while no further increase was obtained with a longer heating time. Additionally, comparison with traditional chemical extraction showed an oil yield around 64% as the highest value. High oil yields were obtained by Głowacz-Rozynska et al. [15] when extracting different by-products (skin, head, and backbone) by three different non-solvent methods (high temperature, 95 °C; low temperature, <15 °C; enzyme-assisted procedure). Thus, oil yields were included in the 71–95% range when compared with traditional extraction. Immanuel et al. [35] studied the oil recovery from Balistid fish (*Sufflamen capistratus*) liver by non-solvent extraction (solar and direct steaming extractions). As a result, green methods led to lower recoveries (round 31 and 40%, respectively) when compared with the one obtained by the chemical-chloroform method (round 70%).

### 2.3. DHA and EPA Recovery from Squid Viscera

The FA analysis of starting viscera oil and oil obtained from pressed viscera revealed a wide range of individual FAs. Thus, the starting viscera oil presented the following FA average composition (g·kg^−1^ oil): 23.5 (C14:0), 3.2 (C15:0), 154.4 (C16:0), 27.2 (C16:1ω7), 6.0 (C17:0), 14.5 (C18:0), 136.9 (C18:1ω9), 23.2 (C18:1ω7), 12.7 (C19:2ω6), 25.5 (C20:1ω9), 4.1 (C20:2ω6), 9.9 (C20:4ω6), 5.3 (C22:1ω9), 92.6 (C20:5ω3), 3.8 (C22:4ω6), 6.2 (C24:1ω9), 5.1 (C22:5ω3), and 164.0 (C22:6ω3). Analysis of the oils obtained from pressed viscera led to the same qualitative composition. On the basis of the above-mentioned nutritional interests, the present study focused on the differences found between the two most important ω3 FAs, i.e., DHA and EPA as well as on the ω3/ω6 ratio.

The presence of DHA (164.0 ± 5.5 g·kg^−1^ oil; 16.4 ± 0.5 g/100 g total FA) and EPA (92.6 ± 1.5 g·kg^−1^ oil; 9.3 ± 0.2 g/100 g total FA) in the lipid fraction of the starting viscera (Table 2) can be considered common to other cephalopod viscera but lower than in the case of edible parts of this biological group [31]. However, previous research has shown that the liver of marine fish and invertebrates is rich in PUFAs [22,28]. Thus, Kacem et al. [27] found similar values to those found in the current study for DHA (9.1 g/100 g total FA) and EPA (11.6 g/100 g total FA) during a seasonal study of cuttlefish (*S. officinalis*) viscera. Contrary, Toyes-Vargas et al. [26] denoted levels of 19.4 and 16.9 g·kg^−1^ viscera in giant squid (*D. gigas*) viscera for DHA and EPA, respectively, which can be considered markedly lower than the current scores detected for both FAs.

In the present study, the content of DHA in the resulting pressed oils was shown to be similar to the starting value in most cases (Table 2). Furthermore, if values are considered on the basis of total FA, the DHA levels in the extracted oils (14.9–16.6/100 g total FA) did not provide differences with the content found in the initial viscera oil. Concerning the effect of the previous drying conditions, the DHA values in the recovered oils were, in all cases, included in a straight range (149.2–166.5 g·kg^−1^ oil). Remarkably, when considering a drying temperature of 65 °C, an increasing effect (*p* < 0.05) on the DHA values by increasing the drying time was found. However, a definite trend for previous drying conditions could not be proved (*p* > 0.05). When considering the DHA content obtained on the viscera basis, great differences were found as a result of the drying conditions; thus, a wide value range was detected (80–13,200 mg·kg^−1^ viscera), the highest DHA content being obtained in samples processed at 85 °C for 90 min. 

As for the DHA level, EPA contents were obtained in a straight range (88.7–102.4 g·kg^−1^ oil; Table 2). When considered on the oil basis (g·kg^−1^ oil), the EPA values in the resulting oil did not show substantial differences with the starting oil (92.6 ± 1.5 g·kg^−1^ oil) regardless of the drying conditions applied. If considered on the basis of the FA composition of the oil, marked differences were also not demonstrated by comparing the EPA range in the extracted oils (8.7–10.2 g/100 g total FA) with the starting oil (9.3 ± 0.2 g total FA). Remarkably, a decreasing average level (*p* < 0.05) was detected in the resulting oil with increasing drying temperature in samples heated for 30 and 90 min, with the differences being significant (*p* < 0.05) when considering samples corresponding to a 90-min drying time. Additionally, increasing average values were obtained in viscera heated at 65 °C by increasing the heating time. However, as for the DHA content in extracted oil, a definite pattern could not be proved (*p* > 0.05) for the previous drying treatment on the EPA level. When the EPA results were considered on a viscera basis, great differences were found as a result of the drying conditions; thus, values were included in the 48–7600 mg·kg^−1^ viscera range, the highest EPA values being obtained in samples processed at 85 °C for 90 min.

Compared with current data, higher levels of DHA (25.5 g/100 g total FA) were obtained by Chantachum et al. [14] during a comparative study on crude oil extraction in precooked and non-precooked skipjack tuna (*K. pelamis*) heads by a wet reduction method; however, negligible levels of EPA were obtained in both kinds of samples. In contrast, higher EPA (15.5 g/100 g total FA) and lower DHA (5.0 g/100 g total FA) levels than in the present study were obtained by Chakraborty and Joseph [10] when applying an extracting method including cooking and wet pressing on edible tissues from Indian sardine (*Sardinella longiceps*). Furthermore, lower EPA (7.1–7.4 g/100 g total FA) and DHA (8.0–8.3 g/100 g total FA) contents were detected in three different salmon (*Salmo salar*) by-products (skins, heads, and backbones) when extracted following three different eco-friendly methods (95 °C for 30 min, <15 °C, and enzyme-assisted, respectively) followed by pressing and centrifugation [15]. Concerning EPA and DHA values, Šimat et al. [12] analysed their presence in different kinds of fish by-products. Thus, the lowest values (g/100 g total FA) were detected in EPA (3.3) and DHA (8.3) both for sea bass (*Sparus aurata*) and sea bream (*Dicentrarchus labrax*) by-products, while the highest levels were found in by-products from tuna (*Thunnus thynnus*) and sardine (*Sardina pilchardus*) (10–14 and 16–21, for EPA and DHA, respectively).

Great attention has been given in the latest decades to the ω3/ω6 FA ratio [36]. Notably, it has been proved that a majority of the Western population do not consume adequate levels of ω3 FA through natural dietary sources. In order to prevent health concerns, such as inflammatory, cardiovascular, and neurological disorders, the European Nutritional Society reported that a human diet with an ω3/ω6 ratio of 1:5 or higher would have health benefits [37]. Furthermore, the World Health Organization (WHO) recommends that this ratio should not be below 1:10 in the human diet [38].

**Figure 1:**
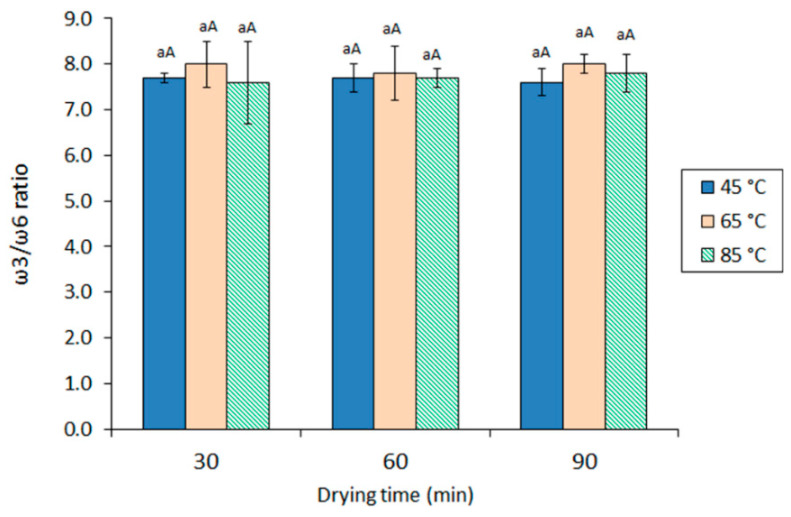
Values for the ω3/ω6 ratio in oil obtained from squid viscera subjected to different drying conditions. Average values of three replicates (*n* = 3); standard deviations are indicated by bars. For each drying time, the same lowercase letter (a) indicates no significant differences (*p* > 0.05) as a result of drying temperature. For each drying temperature, the same capital letter (A) indicates no significant differences (*p* > 0.05) as a result of the drying time. Starting viscera value: 8.6 ± 0.1.

On the basis of such nutritional recommendations, the values obtained in the current study can be considered in all cases as beneficial and highly valuable from a healthy point of view, being included in the 7.6–8.0 range (Figure 1). However, compared with the ratio value of the starting viscera oil (8.6 ± 0.1), a substantial decrease was detected in all extracted oil samples. This result could be explained on the basis that membrane lipids that are not recovered during the extraction process have higher ω3/ω6 ratios. At each drying time, the highest average values were obtained in those samples previously subjected to a 65 °C drying process. Nevertheless, a definite effect of the drying conditions on the ω3/ω6 ratio in the resulting oil could not be concluded (*p* > 0.05).

Different ω3/ω6 values were obtained by Šimat et al. [12] when extracting (cooking at 95 °C for 12 min, pressing, and centrifugation) different kinds of fish by-products. Thus, values included in the 6–10 range were detected for tuna (*T. thynnus*) and sardine (*S. pilchardus*) by-products, while markedly lower ratios (0–2 range) were obtained for tuna (*T. thynnus*) liver and sea bass (*S. aurata*) and sea bream (*D. labrax*) by-products. Lower ω3/ω6 ratio values (5.07 ± 0.02) than in the current study were obtained by Chakraborty and Joseph [10] when extracting edible tissues from Indian sardine (*S. longiceps*) with cooking and wet pressing.

### 2.4. Quality Degree of the Extracted Oil

Lower average values in the free fatty acid (FFA) content were observed in the extracted oils (28.04–37.79 g·kg^−1^) when compared with the FFA level of the starting viscera (Table 3). An increasing drying temperature (*p* < 0.05) led to an increased average FFA level in the extracted oil corresponding to samples dried for 60 min as well as to a decreased average FFA content in samples corresponding to a drying time of 30 min. Therefore, a definite effect of the drying time on the FFA content could not be concluded (*p* > 0.05). Concerning the drying temperature, values observed in the 90-min samples were in all cases higher (*p* < 0.05) than their counterparts corresponding to a drying time of 30 min. As an explanation, the current FFA results can be considered the result of two opposite effects [39,40]. On one side, the heating process can produce hydrolysis development of lipid classes like phospholipids (PLs) and triacylglycerols (TGs), leading to FFA formation. On the other side, FFA are likely to be quickly oxidised or broken down by the heating process to provide greater accessibility to oxygen and other pro-oxidant molecules when compared to TG and PL [41]; therefore, this effect would lead to an FFA content decrease.

Compared with values detected in the edible parts of cephalopod species and marine species in general [31,42], the FFA levels of the current starting viscera can be considered relatively high. Such high values can be explained on the basis of the activity of lipases and phospholipases in visceral tissues [28].

Previous research has considered different FFA ranges for fish oil acceptability. Thus, Sathivel et al. [43] proposed a 1.8–3.5% range for catfish (*Ictalurus punctatus*) oil, while Crexi et al. [44] indicated a 2–5% range for carp (*Cyprinus carpio*) viscera oil. Furthermore, a maximum acceptability at 4% was proposed for salmon (*S. salar*) skin oil [45]. Being below the 3.8% value, the present FFA values for pressed oils can be considered within the limits of acceptability. However, if a lower FFA content is required for further processing or consumption, pressed oils would be susceptible to being subjected to any refining process.

Concerning the conjugated diene (CD) content, scarce differences were detected among the different samples, with all values being included in the 1.06–1.22 range (Table 3). As the most remarkable trends, an increasing drying time led to an increased CD level (65- and 85-°C samples) (*p* < 0.05) and a decreased average CD content was detected by increasing the drying temperature (30- and 90-min samples). Remarkably, the extracted oils showed lower average values than the starting viscera. 

The average values resulting from peroxide determination showed in most cases a decreasing effect by increasing the temperature or time of drying (Table 3). Compared with the starting value, only two of the mildest drying conditions (i.e., drying at 45 or 65 °C for 30 min) led to higher (*p* < 0.05) peroxide levels when compared with the starting oil. In contrast, all other drying conditions led to a substantial decrease of this kind of oxidation compound. Notably, the lowest peroxide value (PV) levels (*p* < 0.05) were found in the oil corresponding to viscera subjected to two of the strongest drying conditions tested (i.e., viscera subjected to 85 °C for 60 or 90 min).

Previous research has reported on the different limits of PV acceptability of oils. Thus, the U. S. Food and Drugs Administration (FDA) suggested that PV should be lower than 2.5 for EPA and DHA oils [46], while Global Optimization for EPA and DHA Omega-3 (GOED) stated that PV should be lower than 5. Concerning refined fish oils, maximum levels of 5 [47,48] and 10 [49] have been proposed for the PV. According to the wide value range obtained in the present pressed oils, subsequent processing or refining could be necessary in some cases to comply with all requirements. However, a lower PV than 10 was detected in most current cases, with such pressed oils being compliant with the British Pharmacopeia Fish Oil Type I, European Union Pharmacopeia Fish Oil Type I, and Australian government guidelines [49,50]. Additionally, the drying conditions leading to the highest oil yield (85 °C for 90 min) provided the lowest PV scores (<1.5).

**Figure 2:**
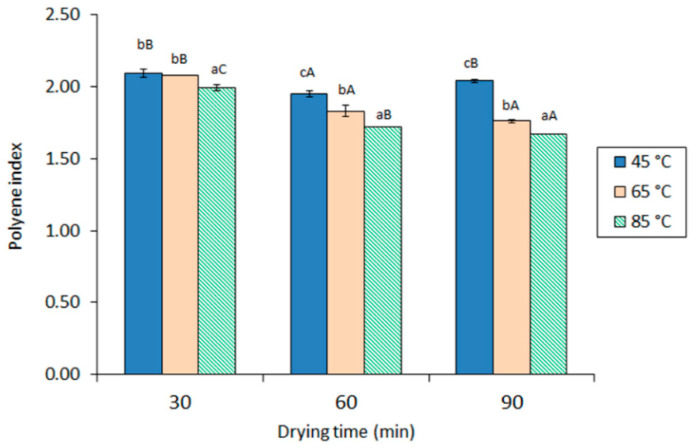
The polyene index in oil obtained from squid viscera subjected to different drying conditions. Average values of three replicates (*n* = 3); standard deviations are indicated by bars. For each drying time, different lowercase letters (a, b, c) denote significant differences (*p* < 0.05) as a result of the drying temperature. For each drying temperature, capital letters (A, B, C) denote significant differences (*p* < 0.05) as a result of the drying time. Starting viscera value: 1.62 ± 0.05.

Concerning the polyene index (PI), a marked effect of drying time and temperature was observed in the extracted oil (Figure 2), with both processing factors leading to lower values (*p* < 0.05). Values ranged between 1.67 (previous drying at 85 °C for 90 min) and 2.09 (previous drying at 45 °C for 30 min), with both being the strongest and mildest conditions tested, respectively. Remarkably, oils from all extracted viscera provided higher average values than the starting viscera (1.62 ± 0.05).

During thermal treatment, such as drying, primary oxidation compounds, such as CD and peroxides, are expected to be produced as a result of oxygen addition to unsaturated FA by a non-enzymatic lipid oxidation mechanism. However, thermal treatment is also reported to inactivate prooxidant enzymes [39], destroy primary oxidation compounds, and lead to low-molecular-weight molecule formation [30,39]. Therefore, the values detected in the current study for both CD and PV determination are the result of both opposite mechanisms. Remarkably, the breakdown of primary oxidation compounds during heat processing has been reported to lead to a loss of PUFA presence in marine lipid substrates [39,40]. Consequently, the observed PI decrease in the current research by increasing the drying time and temperature can be explained on the basis of an increased lipid oxidation in the extracted oil.

Previous research also accounts for an increased lipid oxidation development as a result of heat treatment of by-products. In a comparative study on crude oil extraction in precooked and non-precooked skipjack tuna (*K. pelamis*) heads by a wet reduction method, Chantachum et al. [14] observed markedly higher PV and much darker colour development in precooked samples, when compared with those samples not subjected to precooking. An important effect of heating (higher levels of peroxide and anisidine values) was proved during a comparative extraction of different kinds of by-products of farmed Atlantic salmon (*S. salar*) by non-solvent methods [15]. A marked effect of heating was also observed by Pudtikajorn and Benjakul [11] during oil extraction from skipjack tuna (*K. pelamis*) eyeballs by the wet rendering method at 121 °C for different holding times (5–60 min). Thus, the anisidine value reflected the increased values by increasing the heating time, while peroxide formation revealed a substantial increase up to 30 min but a decrease if applying higher times than 30 min. In contrast, Honold et al. [16] did not find a substantial effect of heating (50–90 °C) when comparatively analysing the effect on oil extraction from heads, bones, tails, and intestines from rainbow trout (*O. mykiss*) filleting. A low formation of peroxides (2–5 meq. active oxygen·kg^−1^ oil) was detected by Šimat et al. [12] when extracting (cooking at 95 °C for 12 min, pressing, and centrifugation) oils of different kinds of fish (tuna, sardine, sea bass, and sea bream) by-products. Furthermore, lipid hydrolysis development led to a 1–3 g FFA/100 g oil range in all cases.

### 2.5. Optimisation of the Process Variables by Means of the RSM

On the basis of the experimental design developed in the current study, an RSM analysis was carried out in order to optimise the processing variables and consequently, values corresponding to the dependent variables. With the same purpose, the RSM has been successfully applied during EPA and DHA urea-concentration from Asian catfish oil (*Pangasius bocourti*) [51], seal blubber (*Phoca groenlandica*) oil [52], and rainbow trout (*Oncorhynchus mykiss*) by-products [53]. Additionally, RSM has also been applied for the optimisation of enzymatic synthesis of acylglycerols including EPA and DHA from salmon (*S. salar*) oil [54] and rainbow trout (*O. mykiss*) by-products [55].

#### 2.5.1. Predictive Second-Order Polynomial Model for the Different Response Variables (Y) and Optimised Response

Table 4 shows the regression coefficients of the predictive second-order polynomial model for the different response variables.

The lipid yield was shown to be significantly affected by the regression coefficients of the lineal terms of the drying temperature (*p* < 0.01; direct relationship) and the quadratic term of the drying time (*p* < 0.04; inverse relationship). The regression analysis results indicated that the coefficient of determination (R^2^ statistic) of the model explained 70.4% of the variability in lipid yield. The adjusted R^2^ statistic, which is more suitable for comparing models with different numbers of independent variables, was 59.3% (Table 4). Concerning other statistic values, the standard error of the estimate (SEE) showed a 22.3 score for the standard deviation of the residuals. A mean absolute error (MAE) value of 16.3 was obtained for the average value of the residuals. Finally, the Durbin–Watson (DW) value was greater than 0.05, so there was no indication of serial autocorrelation in the residuals (*p* > 0.05).

Concerning the PI, the regression coefficients of the linear terms of the drying time (*p* < 0.00) and drying temperature (*p* < 0.00) and the quadratic terms of drying time×drying temperature (*p* < 0.01) and drying time (*p* < 0.00) were highly significant (Table 4). The R^2^ statistic indicated that the model, thus adjusted, explained 95.3% of the variability in PI and the adjusted R^2^ statistic was 92.6% (*p* < 0.05). Meanwhile, the SEE and MAE both showed 0 scores, while DW presented a *p*-value greater than 0.05. 

The peroxide value regression coefficients were significantly affected by the linear terms of the drying time (*p* < 0.00) and drying temperature (*p* < 0.05) and the quadratic term of the drying time (Table 4). The R^2^ statistic indicated that the model, thus adjusted, explained 77.4% of the variability in PV and the adjusted R^2^ statistic was 69.0% (*p* < 0.05). Meantwhile, the SEE and MAE values were 3.3 and 1.9, respectively, and DW presented a *p*-value greater than 0.05.

Table 4 also shows the responses variables for the CD and FFA values. Thus, both variables were significantly affected by the regression coefficient of the drying time linear terms (*p* < 0.05), while drying temperature did not show a significant effect (*p* > 0.05). However, the regression analysis result indicated that the adjusted R^2^ statistic explained only 37.5 and 25.2% of the variability in CD and FFA, respectively (Table 4). For this reason, these response variables were not considered in subsequent multiple-response optimisation by MSR. 

Finally, the DHA and EPA contents were not significantly affected by the regression coefficients of the linear terms, the quadratic terms, or the interaction term (*p* > 0.05) (Table 4). Therefore, it is suggested that the process variables were not determinants of the EPA and DHA content during the current extraction. For this reason, and as for the CD and FFA values, the DHA and EPA contents were not considered in multiple-response optimisation of the response variables by MSR.

#### 2.5.2. Effect of Drying Time and Drying Temperature on Response Variables

**Figure 3:**
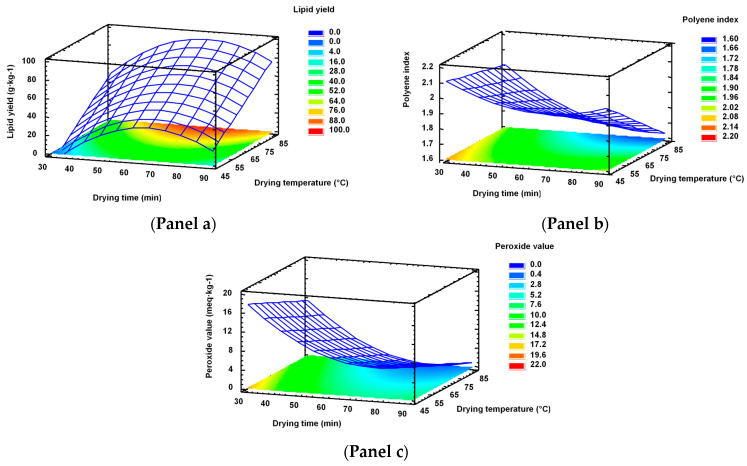
Effect of drying time and drying temperature during oil extraction from squid viscera on the response variables: lipid yield (g·kg^−1^ oil) (**Panel a**), polyene index (**Panel b**), and peroxide value (meq. active oxygen·kg^−1^ oil) (**Panel c**).

The influence of both process variables on the response variables was analysed by RSM (Figure 3). On the basis of the above-mentioned values, only oil yield, PI, and PV were taken into account. Thus, Figure 3 (Panel a) shows the response surface for lipid yield as a function of the drying time and drying temperature. It can be observed that the lipid yield presented a maximum value in the response surface at high levels of the drying temperature and at intermediate levels of the drying time. The optimal lipid yield was 95.99 (Table 4). The optimum combination of factor levels, which maximises the lipid yield over the indicated region, was a drying temperature of 85.0 °C and a drying time of 66.3 min.

Figure 3 (Panel b) exhibits the response surface for the PI. It can be observed that the PI decreased by increasing the drying temperature, while the drying time led to a minimum content of the PI at intermediate levels (*p* < 0.05). The optimum combination of factor levels, which maximises the PI over the indicated region, was 30 min and 45.0 °C and the maximum value obtained of PI was of 2.11.

The peroxide value decreased with the increased drying time (*p* < 0.05) and drying temperature (Figure 3, Panel c). The optimum combination of factor levels, which minimises PV over the indicated region, was 76.4 min and 85.0 °C. Thus, the minimum value obtained of PV was 0.07 (Table 4).

#### 2.5.3. Multiple Response Optimisation of the Response Variables 

**Figure 4:**
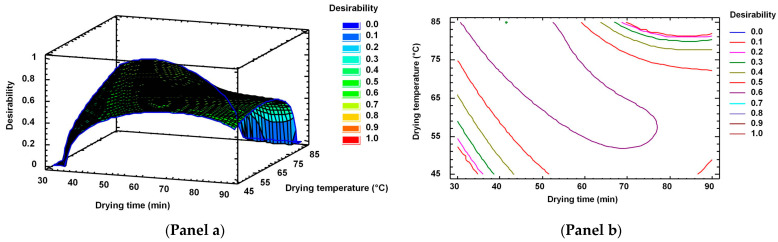
Multiple response optimization of the response variables. Effects of the drying time and drying temperature on the response variables (lipid yield, polyene index, and peroxide value) during oil extraction from squid viscera: desirability function (**Panel a**) and contours of the estimated response surface (**Panel b**).

On the basis of the above-mentioned analysis, only three dependent variables (lipid yield, polyene index, and peroxide value) were considered to calculate the desirability value. Thus, Figure 4 (Panel a and Panel b) shows the contours and estimated response surface of the drying time and drying temperature of the combination of factor levels to maximise the desirability function during oil extraction from squid viscera. The multiple-response optimisation of the different dependent variables was performed using the Derringer desired function methodology [56]. A combined response surface of the optimised response variables was obtained on the basis of the responses obtained for lipid yield, PI, and PV (Figure 4 Panel a). A maximum desirability of 0.67 was obtained in the multiple-response optimisation of oil extraction. The optimal conditions for the drying time and temperature were 41.3 min and 85.0 °C. For such values of independent variables, the responses for the significant dependent variables were 74.73 g·kg^−1^ viscera (lipid yield), 1.87 (PI), and 6.72 (PV).

## 3. Materials and Methods

### 3.1. Raw Material Preparation and Analysis

Fresh squid (*Illex argentinus*) viscera (25 kg) were provided by CEFRICO S. L. (Vilagarcía, Pontevedra, Spain) and transported in ice to the laboratory. Then, 36,500-g portions of viscera were separated, packaged in polyethylene bags, and kept frozen (−80 °C) until use.

On the same day, three 100-g portions of starting viscera (*n* = 3) were analysed for moisture, FA composition, and lipid damage according to the methods described later. For this, the lipid fraction was obtained following the method of Bligh and Dyer [57] in which single-phase solubilisation of the lipids was employed by means of a chloroform–methanol (1:1) mixture. Results on the lipid extraction were calculated as g lipid·kg^−1^ viscera.

### 3.2. Green Extraction of Oil from Squid Viscera

**Figure 5:**
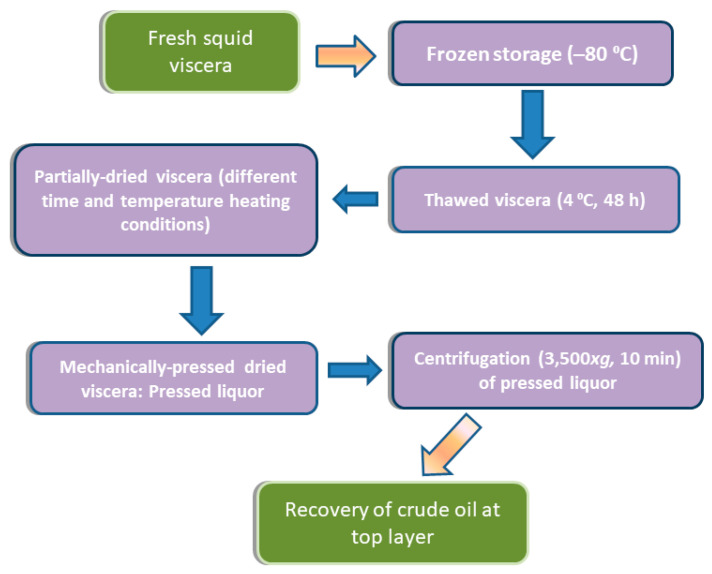
Steps carried out for crude oil recovery from squid viscera by green extraction.

Oil extraction was carried out on viscera previously subjected to a partial moisture reduction consisting of an oven drying process. The whole processing carried out is shown in Figure 5. 

Thus, frozen viscera were thawed under refrigerated conditions (4 °C for 48 h) and subjected to different drying times (30–90 min) and temperatures (45–85 °C) according to the factorial design described in Section 3.6. For this, the following drying treatments were carried out: T-1 (45 °C for 30 min), T-2 (45 °C for 60 min), T-3 (45 °C for 90 min), T-4 (65 °C for 30 min), T-5 (65 °C for 60 min), T-6 (65 °C for 60 min), T-7 (65 °C for 60 min), T-8 (65 °C for 60 min), T-9 (65 °C for 90 min), T-10 (85 °C for 30 min), T-11 (85 °C for 60 min), and 12 (85 °C for 90 min). Treatments were carried out in triplicate (*n* = 3).

Partially dried samples were then subjected to a wet pressing method using a mechanic press. The resulting pressed liquor was then centrifuged at 3500× *g* for 10 min. Finally, the crude oil at the top layer was collected and kept at ‒80 °C until use for analysis.

### 3.3. Determination of Moisture and Oil Yield

Moisture content was determined in the starting viscera and in dried viscera (1–2 g) as the weight difference before and after 4 h at 105 °C [58]. Results were calculated as g·kg^−1^ viscera.

Starting viscera oil and oils obtained from viscera pressing were quantified according to Herbes et al.’s [59] method. Results were calculated as g·kg^−1^ wet viscera.

### 3.4. FA Analysis of Starting Oil and Oil Obtained from Pressed Viscera

Oils were converted into fatty acid methyl esters (FAMEs) by using acetyl chloride in methanol and then analysed by gas-liquid chromatography (GLC; Perkin-Elmer 8700 chromatograph; Madrid, Spain), according to Vázquez et al. [60]. Peaks corresponding to FAMEs were identified by comparing their retention times with those of standard mixtures (Qualmix Fish, Larodan, Malmo, Sweden; FAME mix, Supelco, Inc., Bellefonte, PA, USA). Peak areas were automatically integrated, with C19:0 FA being used as the internal standard for quantitative purposes. Contents of DHA and EPA were calculated as g·kg^−1^ oil and g/100 g total FA and mg·kg^−1^ viscera. Additionally, FAME data were employed for calculating the total ω3/total ω6 ratio. For this, the contents of all ω3 and ω6 FAs were taken into account [42].

### 3.5. Lipid Damage Assessment of Starting Oil and Oil Obtained from Pressed Viscera

The FFA content was determined spectrophotometrically (710 nm; Beckman Coulter DU 640 spectrophotometer, Brea, CA, USA) following the method of Lowry and Tinsley [61]. This method is based on the formation of a complex with cupric acetate-pyridine. Results were calculated as g FFA·kg^−1^ oil.

The PV was determined spectrophotometrically (520 nm) according to the method developed by Chapman and McKay [62] in which peroxides included in the lipid fraction are reduced with ferric thiocyanate. Results were calculated as meq active oxygen·kg^−1^ oil.

CD formation was measured at 233 nm [63] and calculated as CD = A·V·w^−1^, where A is the absorbance reading and V and w are the volume (mL) and weight (mg), respectively, of the oil aliquot taken for analysis.

The PI was calculated on the basis of the above-mentioned GLC analysis of FAME as the following FA content ratio [60]: (C20:5ω3 + C22:6ω3)/C16:0.

### 3.6. Optimisation of Response Variables

The study was performed following a factorial design of 3^2^ of 12 experiments based on the RSM. Three replicates were performed at the central point to estimate the experimental error. The conditions for the process variables included the drying time (from 30 to 90 min) and drying temperature (from 45 to 85 °C) as expressed in Section 2.2. The response variables of the experiment design included the lipid yield, PI, PV, and contents of DHA, EPA, FFA, and CD. 

All experiments were carried out randomly to minimise the effect of unexplained variability in the observed responses due to extraneous factors. From RSM, a mathematical model was obtained, with which the effect of the fixed independent variables could be predicted. Regression coefficients were obtained by multiple regression analysis considering a significance level of *p*-value < 0.05. A quadratic polynomial regression model was assumed for predicting the individual Y dependent variables [64,65]. The model proposed for each response of the Y value is expressed in the following equation:

(1) $$Y_{i}=\beta_{0}+\sum_{i=1}^{2}\beta_{i}X_{i}+\sum_{i=1}^{2}\beta_{\mathrm{ii}}X_{i}^{2}+\sum_{\;i=1}^{1}\sum_{\;j=i+1}^{2}\beta_{\mathrm{ij}}X_{i}X_{j}+\varepsilon$$

where β_0_, β_i_, β_ii_, and β_ij_ represent the intercept, linear, quadratic, and interaction regression coefficients, respectively; X_i_ and X_j_ represent the independent variables; and ε corresponds to the experimental error [66]. A multiple-response optimisation was performed to optimise several responses simultaneously, with this maximising the desirability function that ranged between 0 and 1 scores [66]. The statistical program Statgraphics Centurion XVI-2011 (StatPoint Technologies, Inc., Rockville, MD, USA) was used.

### 3.7. Statistical Analysis

Data corresponding to the moisture loss of dried viscera, liquor weight released after pressing, and extracted oil from viscera (yield, FA composition, and damage degree) were subjected to one-way ANOVA to explore differences resulting from the drying conditions (time and temperature).

ANOVA analysis was performed to obtain significant differences of the independent variables with *p*-value < 0.05 and multiple regression analysis to obtain the adjusted quadratic polynomial models of the response variables studied. In addition, the estimated response surface and contour surface were obtained for each of the dependent variables in order to identify the effect of each of the independent variables.

In all cases, the confidence interval used was 95%, taking into account the number of replications and considering the standard deviation of each sample. The statistical program Statgraphics Centurion XVI-2011 (StatPoint Technologies, Inc., Rockville, MD, USA) was used.

## 4. Conclusions

In general, an increase of the drying time and temperature provided an increase of the lipid yield recovery from viscera; thus, the strongest drying conditions showed a recovery higher than 50% when compared with the lipid recovery by the traditional chemical method. The DHA and EPA contents in oil revealed scarce dependence on the drying conditions, leading to valuable ranges (149.2–166.5 and 88.7–102.4 g·kg^−1^ oil, respectively). Furthermore, the values of FFA, peroxides, CD, and ω3/ω6 ratio did not show extensive differences by comparing oils obtained from the different drying conditions. In contrast, a PI decrease was detected with increasing drying conditions. The multiple-response optimisation revealed a maximum desirability of 0.67. Thus, the RSM analysis indicated that the optimised drying time (41.3 min) and temperature (85 °C) conditions would lead to 74.73 g·kg^−1^ (oil yield), 1.87 (PI), and 6.72 (PV) scores, with a 0.67 desirability value. Such values can be considered as being compliant with international requirements of marine oil quality. 

The present green extraction matches with current global interests in the search for alternatives for oil extracting methods reducing the risk of chemical exposure to humans and the environment. Furthermore, the present study contributes to the achievement of alternative sources of ω3 PUFAs from discarded by-products, with the aim of increasing the profitability of such by-products and the suitability of finding ω3 PUFA sources, preventing overfishing of common species. The current results show that oil extraction by green processing is an interesting option for the recovery of high-value compounds, such as EPA and DHA, from squid (*I. argentinus*) viscera, as well as a valuable ω3/ω6 ratio. Additionally, the employment of an RSM design can provide the possibility of affording a product with, at the same time, a minimum rancidity level and maximum oil yield and polyene index. 

## Figures and Tables

**Table 1:** Effect of the preliminary drying conditions * on the moisture loss of squid viscera (g·kg^−1^ viscera) and on the pressed liquor yield (g·kg^−1^ viscera) **.

	Drying Time (min)	Drying Temperature (°C)		
		45	65	85
Moisture loss	30	1.7 aA (0.4)	3.9 bA (0.9)	9.0 cA (3.2)
	60	9.1 aB (2.1)	17.2 abB (6.7)	28.0 bB (7.6)
	90	8.7 aB (1.6)	26.0 bB (7.5)	51.1 cC (10.3)
Pressed liquor yield	30	308.2 aA (30.5)	341.8 aA (39.0)	355.0 aA (40.1)
	60	410.5 aB (32.1)	535.7 bB (67. 8)	641.4 bB (72.0)
	90	279.4 aA (28.8)	616.1 bB (71.4)	598.4 bB (64.5)

* Average values of three replicates (*n* = 3); standard deviations are indicated in brackets. For each drying time, different lowercase letters denote significant differences (*p* < 0.05) as a result of the drying temperature. For each drying temperature, capital letters denote significant differences (*p* < 0.05) as a result of the drying time. ** Moisture value of the starting viscera: 647.3 ± 4.4 g·kg^−1^ viscera.

**Table 2:** Oil yield and docosahexaenoic (DHA) and eicosapentaenoic (EPA) acid values * obtained from squid viscera subjected to preliminary drying conditions **.

	Drying Time (min)	Drying Temperature (°C)		
		45	65	85
Oil yield (g·kg^−1^ viscera)	30	0.5 aA (0.2)	1.1 aA (0.2)	59.9 bA (1.2)
	60	1.0 aA (0.2)	79.4 cC (7.1)	66.0 bB (2.8)
	90	25.2 aB (2.8)	29.3 aB (4.8)	84.1 bC (1.0)
DHA content (g·kg^−1^ oil)	30	159.9 aAB (8.3)	149.6 aA (2.6)	153.5 aA (5.7)
	60	149.2 aA (2.1)	158.6 aB (3.9)	149.5 aA (3.6)
	90	166.3 bB (0.1)	166.5 bC (2.1)	156.9 aA (4.6)
EPA content (g·kg^−1^ oil)	30	96.3 aA (4.8)	93.0 aA (1.2)	92.4 aA (3.4)
	60	91.3 aA (0.7)	93.3 aAB (4.0)	88.7 aA (2.2)
	90	102.4 cB (0.3)	98.5 bB (1.3)	90.8 aA (2.7)

* Average values of three replicates (*n* = 3); standard deviations are indicated in brackets. For each drying time, different lowercase letters denote significant differences (*p* < 0.05) as a result of the drying temperature. For each drying temperature, capital letters denote significant differences (*p* < 0.05) as a result of the drying time. ** Starting viscera values: 111.3 ± 6.3 (oil content; g·kg^−1^ viscera), 164.0 ± 5.5 (DHA; g·kg^−1^ oil), 18.3 ± 0.7 (DHA; g·kg^−1^ viscera), 92.6 ± 1.5 (EPA; g·kg^−1^ oil), and 10.3 ± 0.5 (EPA; g·kg^−1^ viscera).

**Table 3:** Lipid damage assessment * of oil obtained from squid viscera subjected to preliminary drying conditions **.

	Drying Time (min)	Drying Temperature (°C)		
		45	65	85
Free fatty acid (FFA) content (g·kg^−1^ oil)	30	32.15 bB (1.12)	28.99 aA (1.14)	28.04 aA (0.90)
	60	30.52 aA (0.11)	32.16 aB (1.90)	35.51 bB (0.96)
	90	37.79 cC (0.34)	31.08 aB (0.68)	34.67 bB (0.34)
Conjugated dienes (CD) value ***	30	1.12 aAB (0.06)	1.10 bA (0.00)	0.99 aA (0.05)
	60	1.08 aA (0.01)	1.12 aAB (0.08)	1.06 aAB (0.05)
	90	1.22 bB (0.04)	1.21 bB (0.01)	1.11 aB (0.01)
Peroxide value (PV; meq active oxygen·kg^−1^ oil)	30	17.76 bC (1.25)	19.85 bB (1.93)	6.69 aB (0.91)
	60	3.14 bA (0.91)	5.62 bA (1.85)	1.85 aA (0.23)
	90	7.42 cB (1.94)	3.54 bA (0.80)	1.44 aA (0.34)

* Average values of three replicates (*n* = 3); standard deviations are indicated in brackets. For each drying time, different lowercase letters denote significant differences (*p* < 0.05) as a result of the drying temperature. For each drying temperature, capital letters denote significant differences (*p* < 0.05) as a result of the drying time. ** Starting viscera values: 38.67 ± 1.91 (FFA), 1.24 ± 0.12 (CD), and 13.25 ± 3.63 (PV). *** Units as expressed in the Materials and Methods section.

**Table 4:** Regression coefficient values * of the predictive second-order polynomial model for the different response variables (Y) **.

Response Variables (Y)	Process Variables **						Optimised Response	
	β_0_	β_1_ X_1_	β_2_ X_2_	β_11_ X_1_^2^	β_12_ X_1×_X_2_	R^2^		
		(min)	(°C)	(min^2^)	(min × °C)	(%)	Stationary point	Optimum value
Lipid yield	–183.93	4.53 (0.20)	1.53 (**0.01**)	–0.03 (**0.04**)	-	**59.3**	Maximum	95.99
Polyene index	2.44	–0.01 (**0.00**)	0.01 (**0.00**)	0.00 (**0.00**)	–0.00 (**0.01**)	**92.6**	Maximum	2.11
Peroxide value	44.62	–0.83 (**0.00**)	–0.15 (**0.05**)	0.01 (**0.03**)	-	**69.0**	Minimum	0.07
EPA content	10.03	0.01 (0.30)	–0.02 (0.08)	-	-	22.4	Maximum	9.86
DHA content	15.67	0.02 (0.08)	–0.01 (0.27)	-	-	23.4	Maximum	16.40
Conjugated dienes	1.12	0.00 (**0.04**)	–0.00 (0.15)	-	-	37.5	Minimum	1.21
FFA content	28.71	0.08 (**0.04**)	–0.02 (0.72)	-	-	25.2	Minimum	29.51

* β_0_, β_i_, β_ii_, β_ij_ represent the constant, linear, quadratic, and interaction regression coefficients, respectively; X_1_ (drying time) and X_2_ (drying temperature) represent the independent variables. R^2^ values adjusted by freedom degree. ** For each regression coefficient, the *p*-value is indicated in brackets. Significant values (*p* < 0.05) are marked in bold print. Adjusted R^2^ values corresponding to the dependent variables affected by both process variables are marked in bold print. The units of the response variables as expressed in Table 2 and Table 3.
